# Intensive Care in Traumatic Brain Injury Including Multi-Modal Monitoring and Neuroprotection

**DOI:** 10.3390/medsci7030037

**Published:** 2019-02-26

**Authors:** Reto A. Stocker

**Affiliations:** Institute for Anesthesiology and Intensive Care Medicine, Klinik Hirslanden, CH-8032 Zurich, Switzerland; reto.stocker@hirslanden.ch

**Keywords:** traumatic brain injury, treatment, neuro intensive care, multimodal monitoring

## Abstract

Moderate to severe traumatic brain injuries (TBI) require treatment in an intensive care unit (ICU) in close collaboration of a multidisciplinary team consisting of different medical specialists such as intensivists, neurosurgeons, neurologists, as well as ICU nurses, physiotherapists, and ergo-/logotherapists. Major goals include all measurements to prevent secondary brain injury due to secondary brain insults and to optimize frame conditions for recovery and early rehabilitation. The distinction between moderate and severe is frequently done based on the Glascow Coma Scale and therefore often is just a snapshot at the early time of assessment. Due to its pathophysiological pathways, an initially as moderate classified TBI may need the same sophisticated surveillance, monitoring, and treatment as a severe form or might even progress to a severe and difficult to treat affection. As traumatic brain injury is rather a syndrome comprising a range of different affections to the brain and as, e.g., age-related comorbidities and treatments additionally may have a great impact, individual and tailored treatment approaches based on monitoring and findings in imaging and respecting pre-injury comorbidities and their therapies are warranted.

## 1. Background

Traumatic brain injury (TBI) still is a major cause of death and disability worldwide, with more than 13 million people estimated to live with disabilities related to TBI in Europe and the USA. In developed countries, TBI increasingly affects people older than 65 years, typically after falls from low height, while the number of patients aged 15–44 years as, e.g., victims of high velocity traffic accidents has decreased due to improved road conditions, stronger enforcement of traffic regulations, and improved safety features of vehicles [1]. The burden of TBI in younger patients becomes more and more prominent in low- and middle-income countries, which face a higher preponderance of risk factors for causes of TBI and have inadequately prepared health systems to address the associated health outcomes. Latin America and Sub-Saharan Africa demonstrate a higher TBI-related incidence rate varying from 150–170 per 100,000 due to road traffic accidents compared to a global rate of 106 per 100,000. About 10–15% of patients with TBI have serious injuries that require specialist care, primarily in an intensive care unit (ICU) [2].

The combined medical–surgical approach has changed little over the past 20 years, although the scientific evidence supporting most interventions is weak [3]. Therefore, clinicians have to rely on expert opinions, based on decades of accumulated and refined clinical experience [4]. Furthermore, treatment guidelines frequently applied to all patients are usually derived from cohort studies. This approach ignores differences in underlying pathological features and influence of pre-injury conditions. TBI is rather a syndrome including a range of brain lesions with often diverging pathophysiological pathways, which additionally is modified by patient-related pre-injury factors. Therefore, therapeutic needs may vary from patient to patient and should be targeted to the specific pathophysiological mechanisms of the individual patient. Moreover, current knowledge about TBI has mainly been generated from young (typically male) patients suffering from high-velocity traffic injuries or assaults. However, in developed countries, TBI increasingly affects people older than 65 years, typically after falls from low height, while incidence for patients aged 15–44 years has declined [1]. This adds risk factors typical of elderly people, such as age-related comorbidities and their pharmacological treatment. 

## 2. Pathological and Pathophysiological Features

**Figure 1:**
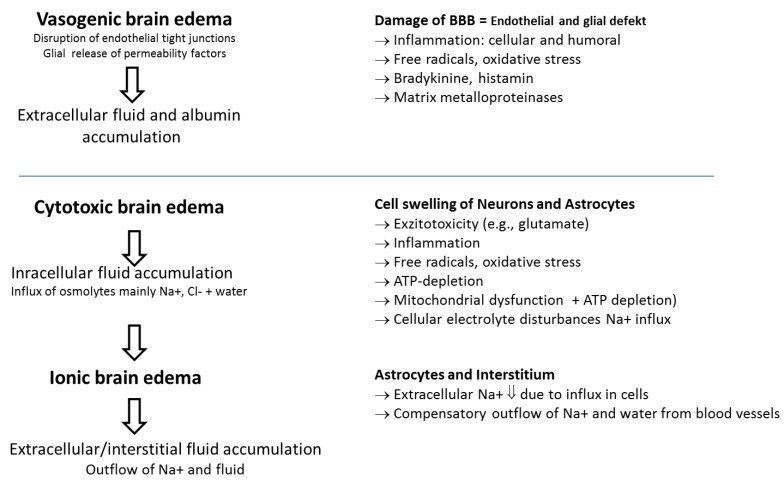
Description of etiology, cause, and consequences of brain edema/brain swelling. Adapted from Michinaga, S. *Int. J. Mol. Sci*. **2015** [13].

The magnitude of damage to cerebral tissues following head trauma is determined by primary and delayed secondary injuries. Primary injuries are caused by the kinetic energy delivered by external physical forces (blows, acceleration/deceleration, and rotation) at the time of impact, resulting in skull fractures, hematomas, and deformation and destruction of brain tissue, including contusions and axonal injury. Numerous secondary injuries that almost inevitably worsen the primary injury develop over time with activation of multiple molecular and cellular pathways and may be promoted by secondary insults (e.g., hypotension, hypoxia, hypocarbia, hyperglycemia, hypo-/hyperthermia, electrolyte disorders, epileptic seizures exacerbating the imbalance between energy expenditure and supply) [5,6]. Moreover, spreading depolarization, another electrical disturbance leads to anaerobic metabolism, energy substrate depletion, and also seems to be associated with a worse outcome. Axonal stretching during injury potentially also leading to synaptic disconnection, among others, can disrupt the myelin sheath with impairment of signal transduction (simultaneous firing neurons). This induces dysregulation of transmembrane ion fluxes (potassium efflux and calcium influx) and may impair axonal transport and consecutively increases vulnerability to secondary axotomy and demyelination. Changes in ionic permeability and release of excitatory neurotransmitters, particularly glutamate, propagate damage through energy failure and increase calcium influx into the cell, leading to intracellular calcium overload and overload of free radicals. Calcium overload causes mitochondrial dysfunction, triggering further energy defects and necrotic and apoptotic processes [7]. All these changes may promote the development of cytotoxic and/or vasogenic (increased permeability of the blood–brain barrier (BBB)) brain edema and may impair autoregulation (Figure 1). According to the Monro–Kellie doctrine, the sum of the volumes of the intracranial contents (vessels, cerebral blood volume (CBV), cerebrospinal fluid (CSF), brain tissue, and mass lesions) remains constant due to the encapsulating skull [8]. Therefore, increases in CBV resulting from vasodilation (vascular engorgement) and/or increase in extravascular water accumulation due to a disruption of the BBB (vasogenic brain edema) and/or cell swelling as well as non-evacuated mass lesions go on the expenses of CSF and tissue volumes (compression) [9]. Once this volume increase exceeds the compensatory capacities of the intracranial space (e.g., decrease of CSF volume), intracranial pressure (ICP) rises.

Trauma leads to activation of a proinflammatory state (systemic inflammatory response syndrome (SIRS) [9]. Neuroinflammation, also promoted by resident microglia, may aggravate secondary injury [10]. Moreover, concomitant extracranial injuries and massive bleeding may cause hypoxia or arterial hypotension and thus promote SIRS that can further aggravate the development of secondary injury [11]. This complex series of events starts minutes after trauma but lasts for weeks or even months, particularly for inflammation [12].

## 3. Traumatic Brain Injury Is Not a Single Entity

Traumatic brain injury produces various lesions that range from mild to devastating injury. Initial classification typically is based on clinical severity using the Glasgow coma scale (GCS). A severe TBI shows a score of 8 or less [14]. Space occupying and/or expanding epi-, subdural, and intracerebral hematomas might require emergency surgical evacuation in the first hours after injury. When head trauma results in a cerebral contusion/intracerebral hematoma, these lesions progress in more than 50% during the first several hours after impact, either expanding or developing new, noncontiguous hemorrhagic lesions. This phenomenon is termed hemorrhagic progression of a contusion (HPC) [15]. Because a hemorrhagic contusion occurs in tissues with unrecoverable loss of function, and because blood is very toxic to the brain, HPC is one of the most severe types of secondary injury encountered following TBI. In the past, HPC mainly had been attributed to persistent bleeding of microvessels disrupted at the time of primary injury. This suggests that continued bleeding might be due to overt or latent coagulopathy, prompting attempts to normalize coagulation with agents such as recombinant factor VIIa (rFVIIa). Recently, a different mechanism was postulated to be responsible for HPC, possibly explaining why rFVIIa in early management of TBI could not be shown to be associated with a decreased risk of mortality or morbidity [16]. This involves delayed, progressive microvascular failure in the region of injury (penumbra) initiated by an impact that is not sufficient to disrupt the microvessels but is sufficient to induce a series of maladaptive molecular events, eventually resulting in their structural failure. Consecutively, this leads to delayed formation of petechial hemorrhages, which then merge to produce hemorrhagic progression, eventually sometimes needing surgery as well [17]. More subtle lesions (e.g., traumatic axonal injury or diffuse axonal injury when involving three or more locations might not be evident from the initial CT or even follow-up scans but can be seen on magnetic resonance imaging (MRI)) [18]. However, owing to neuronal network disruption by disruption of neuronal connectivity, they might have serious consequences for the quality of life of survivors [19].

The different lesion types typically may occur in combination. While brain contusions, intracerebral, and subdural hematomas, and fast growing non-evacuated epidural hematomas bear a high risk for high ICP and consecutive severe disability or mortality, the risk for ICP increase is low for axonal injury, traumatic subarachnoidal hemorrhage and petechial bleedings [19,20]. Nevertheless, risk for disability and mortality mainly for axonal injury in multiple brain regions but also for extensive traumatic subarachnoidal hemorrhage may be high, too [21]. 

In contrast to younger patients, older patients typically suffer from low-energy impacts (e.g., ground-level falls), leading to a higher proportion of subdural hematomas and fewer contusions or epidural hematomas [22,23,24]. On the other hand, cerebral atrophy and increased CSF space buffers space occupying intracranial masses, resulting in a lower incidence of raised ICP [25]. Moreover, age-related comorbidities with reduced physiological reserves to cope with, e.g., acute phase reaction, neurohumoral and metabolic changes, and the fact that post-traumatic seizures are more common in older patients increase incidence and severity of early secondary insults (especially hypoxia and hypotension), promoting secondary brain damage. 

## 4. Multimodal Monitoring and Treatment of Patients with Moderate to Severe TBI

Patients with severe TBI are currently treated in the ICU with a specialized neurointensive approach combined with strategies used in general intensive care, such as fluid optimization and use of vasoactives and catecholamins for arterial pressure and splanchnic organ perfusion, artificial ventilation aiming at normalization of respiratory gas exchanges, temperature control, infection control and treatment, and early enteral feeding by a multiprofessional team including intensivists in close collaboration with neurosurgeons, neurologists, skilled nurses, physiotherapists, and rehabilitation specialists. This approach aims to prevent second insults, maintain cerebral homoeostasis, and preserve rehabilitation potential. 

### 4.1. Neurological Clinical Examination and Early Imaging

Clinical examination remains a fundamental monitoring procedure, even in patients who are comatose or sedated, to identify neurological deterioration and potential indications for surgical interventions. The basic examination relies on a GCS assessment coupled with investigation of pupil diameter and reactivity to light. However, GCS assessment is frequently performed on the site of the accident and often does not/cannot respect the precondition of being resuscitated according to the original description of GCS assessment [26]. Therefore, GCS calculated and forwarded by the emergency team may overestimate degree of disturbance of consciousness. As tracheal intubation precludes a verbal response and eye opening may be compromised by facial injuries, motor response remains the main assessable component of the GCS score. Therefore, the motor component of the Glascow coma score is valued as the most robust and important part of GCS evaluation.

Neurological evaluation in patients who are deeply sedated would require interruption of sedation (wake-up test), which might cause deterioration, e.g., arterial hypertension, fighting the ventilator, increased oxygen consumption, and transient rises in ICP [27]. To what extent this might be detrimental for brain homoeostasis is not absolutely clear [28]. Nevertheless, a wake-up test may help to identify important clinical changes (deterioration or improvement) and could influence management with more aggressive intervention in patients who show deterioration or shorter intubation and ventilation times in those recovering favorably.

Assessments of pupillary diameter and reactivity are crucial [29]. A dilated unreactive pupil usually results from compression of the third cranial nerve (e.g., midline shift and uncal herniation). To date, automated pupillometry, a portable technique that measures pupil size and light reactivity, automatically improves inter-rater accuracy, particularly when the pupil is small (e.g., with opioid analgesia) [30]. 

More than 40% of patients with TBI show substantial worsening during the first 48 h in the ICU as a decrease of ≥2 points on GCS motor component, pupil asymmetry or loss of pupillary reactivity, or deterioration in neurological or CT status, sufficient to warrant immediate medical or surgical intervention. This is significantly associated with high ICP and poor outcome [31].

Among others, neurological worsening also results from prompt access to early CT (within minutes after TBI) before lesions have had a chance to appear or evolve. As mentioned above, parenchymal lesions can expand or newly appear over hours or days. A routine second CT scan within 24 h should therefore be performed in all patients who are comatose. This might show lesions requiring surgery in up to a third of cases [32]. Additionally, if any substantial clinical worsening occurs or ICP rises, a new CT scan must be done.

### 4.2. Intracranial Pressure and Cerebral Perfusion Pressure

#### 4.2.1. Intracranial Pressure

A significant number of patients with severe TBI develop raised ICP, generally depending on intracranial lesion, age, and definition. The most widely accepted ICP threshold for therapy is 20 mmHg, although the latest guidelines suggest 22 mmHg [2]. However, as mentioned above, this general threshold limits an optimal therapy according to the needs of individual patient. Available data suggest that critical ICP thresholds may vary between young and old and male and female patients, with older patients (age ≥55 years) and females having lower ICP thresholds (18 mmHg vs. 22 mmHg) for prediction of poor outcome [33].

Intracranial pressure monitoring has been the cornerstone of TBI care since the 1980s. Devices used in adults include subdural or intraparenchymal probes or ventricular catheters connected to a monitor. Even though a multicenter trial did not show superiority of management using continuous measurement of ICP compared to repeated clinical examination and CT scans, the role of ICP monitoring should not be abandoned, as the study mainly highlighted that there is no direct link between monitoring alone and improvement of outcome [34]. 

According to the actual guidelines, ICP monitoring is indicated in patients with severe TBI (GCS ≤8). This recommendation is based on some evidence suggesting that ICP-guided treatment can reduce early mortality [35]. 

Protocols for ICP therapy vary in detail and are mainly based on experience and consensus guidelines but not on clear scientific evidence. In principle, there are three main strategies proposed by the scientific community: A traditional approach targeting treatment of elevated ICP, the Rosner concept aiming at control of cerebral perfusion pressure (CPP), and the Lund concept, a volume-targeted treatment approach.

#### 4.2.2. Intracranial Pressure Guided “Traditional” Approach

Continuous monitoring of ICP has become the cornerstone in neuromonitoring, as it reflects predisposition to cerebral injury and herniation. Although there are no randomized trials confirming the benefit of ICP monitoring and treatment in TBI, substantial support that ICP monitoring improves outcome can be found in the literature [36,37,38,39]. The “traditional” approach to treat an elevated ICP (proposed threshold 20 mmHg) with head elevation, sedation, active treatment of systemic hypertension, neuromuscular blockade, cerebrospinal fluid (CSF) drainage usually by external ventriculostomy, osmotherapy, hyperventilation, and induction of a barbiturate coma to reduce and control intracranial pressure [40] has mainly been abandoned, as it neglects the detrimental effects of hyperventilation, lowering blood pressure, head elevation, osmotherapy, and barbiturates (Table 1). Moreover, some studies have indicated that despite extremely high ICP, intense, aggressive management of CPP can lead to good neurological outcomes [41]. 

#### 4.2.3. Cerebral Perfusion Pressure Guided Therapy 

Oxygen delivery to the brain is dependent on different factors, such as cerebral blood flow (CBF) and oxygen content of the blood. CBF itself again is dependent on cardiac output, cerebral perfusion pressure (CPP; difference between mean arterial blood pressure and ICP) and cerebrovascular resistance (vasoregulation, e.g., autoregulation, hypocapnic vasoconstriction, and vasocompression, e.g., due to elevated intracranial pressure). In patients with maintained autoregulation, targeting CPP as surrogate for relative cerebral perfusion is more important than just targeting an increased ICP. However, considerable uncertainty exists about the optimal level of CPP. In order to approach this issue, it is important to keep in mind that cerebral oxygenation depends on oxygen delivery and oxygen consumption (cerebral metabolic rate of oxygen (CMRO_2_)). CPP as a surrogate for CBF in the healthy brain is defined as the difference between mean arterial pressure (MAP) and the pressure in the small cerebral veins just before they enter the dural sinuses. Thus, CPP represents the driving pressure for CBF and, thus, oxygen and metabolite delivery. In a normal brain, cerebral blood flow over a wide range is autoregulated to provide a constant blood flow regardless of changes in blood pressure by altering the resistance of cerebral blood vessels (cerebrovascular resistance (CVR)). Therefore, CVR is high when intravascular pressure is high, and CVR is low when intravascular pressure is low. Cerebrovascular resistance is controlled by myogenic, metabolic, and neurogenic factors whose interactions at present are still poorly understood. Although the classic description of the CBF pressure autoregulation is widely accepted, a significant variation in the intersubject pattern or shape of the circulatory response to hypotension has been reported [42]. The homeostatic mechanisms are often at least partially lost after head trauma (CVR is usually increased), and the brain becomes more susceptible to blood pressure changes and systemic hypotension, then further aggravates cerebral ischemia [43]. Additional factors that may impair autoregulation include hypoxemia, hypercapnia. and hypocapnia, as CBF normally decreases in response to hyperventilation. This CO_2_ reactivity is usually, but not always, preserved following head injury. Either a decrease in MAP or an elevation in ICP will deleteriously alter the effective CPP. Historically, maintenance of a CPP greater than 50 mmHg was considered acceptable in head injury patients. However, following trauma, if autoregulation is preserved, it is shifted to the right; therefore, a higher CPP is needed to maintain adequate CBF. Increasing CPP also minimizes ICP by reducing cerebral blood volume (CBV) and cerebral edema via autoregulatory vasoconstriction. Rosner et al. recommended maintaining a CPP greater than 70 mmHg in head-injured patients to minimize cerebral ischemia and prevent the cascade of events that result from inadequate perfusion [44]. However, as autoregulation is often impaired, an increase in arterial pressure may lead to an increase in CBF and CBV and, therefore, ICP. Moreover, increased capillary hydrostatic pressure may increase cerebral edema and intracranial hypertension. This part of pathophysiology is the main tenet of the so called “Lund” approach (see below) [45]. 

#### 4.2.4. Rationale for Cerebral Perfusion Pressure Directed Therapy in Traumatic Brain Injuries

Clinical use of CPP is based on the theory that optimal cerebral blood flow (CBF) is necessary to meet the metabolic needs of the injured brain. The goal is to preserve the ischemic penumbra and avoid exacerbation of secondary insults, such as excitotoxicity, free radical production, and inflammation. Rosner and Daughton have shown that a CPP above 70 mmHg may improve patient outcome [46]. Therefore, the concept of prophylactically elevating CPP to avoid brain ischemia and to maintain an ideal CBF for a while has gained support. Subsequently, a randomized controlled trial demonstrated that maintenance of CPP higher than 70 mmHg was associated with five times greater risk of acute respiratory distress syndrome (ARDS) [47]. Moreover, CPP values higher than 70 mmHg did not offer any outcome benefits [48]. Accordingly, it has been recommended that a CPP above 70 mmHg should be avoided. However, a low CPP causes reduction in CBF, may induce an elevation in CBV and thus ICP within the range of autoregulation, and predisposes the injured brain to cerebral ischemia and infarction [49,50]. 

Studies using microdialysis, jugular venous oximetry, and brain tissue oxygen saturation (PbtO_2_) demonstrated signs of ischemia with a CPP below 50 mmHg, while a CPP above 60 mmHg could help to avoid cerebral oxygen desaturation [51,52,53]. This suggests that the critical threshold for CPP lies between 50 and 60 mmHg [54]. However, as cerebral blood flow and metabolism are heterogeneous after TBI and with regional temporal differences in the requirement for CPP, brain monitoring techniques such as jugular venous oximetry (SvJO_2_), monitoring of brain tissue oxygen tension (PbtO_2_), and cerebral microdialysis could provide complementary and specific information that allows for selecting the optimal CPP for the individual patient. Among others, optimization of cerebral oxygen supply can be guided by the CPP. A decreased MAP should be corrected using vasopressors and fluid expansion. Goal MAP has to take into consideration that autoregulation—if preserved—is subjected to a right shift in patients with preexisting hypertension. However, this does not respect individual needs and does not consider that ICP in the different compartments within the skull might be different. Maintenance of a certain CPP threshold makes sense only if autoregulation in general is preserved, as otherwise, MAP and ICP might be parallel [54]. The latest guidelines therefore suggest discrimination between individuals with and without preserved autoregulation [2].

The CPP-targeted treatment concept includes:

1. Prevention of ICP rises and maintenance of CPP by basic measures:
  - Analgosedation;
  - Normocapnic mechanical ventilation;
  - Normovolemia using normo-osmolar balanced crystalloids or even colloids (no albumin [55,56];
  - Mean systolic arterial pressure of ≥100 mmHg;
  - Maintenance of a cerebral perfusion pressure of 60–70 mmHg if autoregulation is preserved;
  - Maintenance of a normal body temperature;
  - Normoxemia;
  - Enteral feeding,
2. If ICP increases, active interventions might be warranted:
  - Deepening analgosedation;
  - Detection and treatment or exclusion of surgically accessible space occupying lesions with imaging;
  - Drainage of cerebrospinal fluid (insertion of a ventricular catheter if ventricles are not already fully compressed; risk of infection);
  - Increase of mean arterial pressure if autoregulation is preserved in order to reduce cerebral blood volume without compromising cerebral blood flow (risk of cardiovascular side effects, risk of promoting edema formation);
  - Use of hyperosmolar solutions (NaCl 3–7.5% (HTS; hypertonic saline), Na–lactate 1100 mosm/L, Mannitol 20%;
    Hyperosmolar agents are effective in reducing ICP. What remains unanswered is whether these agents contribute toward better neurological outcomes; tiered, algorithmic approaches employed in many trials make it difficult to determine which therapy is beneficial or potentially harmful. Mannitol seems to be less effective than NaCl 7.5% and hypertonic Na–lactate in reducing brain swelling after head injury. Moreover, there is evidence that excessive administration of mannitol may be harmful, because mannitol might pass from the bloodstream into the brain, where it worsens brain edema and increases ICP (rebound edema). In contrast to NaCl 7.5%, mannitol does not improve tissue oxygenation [57]. While both osmolar agents increase cerebral blood flow, the magnitude of augmentation seems to be greater in HTS treatment and HTS seems to reduce the accumulation of extracellular excitatory amino acid (glutamate), thus preventing glutamine toxicity and neuronal damage [58]. However, there is no difference in neurologic outcome between the treatments at 6 months using the Glasgow outcome score [59]. In general, evidence for improved neurological outcome for all these interventions is still very limited, and they bear a risk of fluid overload including cardiovascular events and risks of osmotic diuresis, including dehydration [60].
3. Aggressive treatment of refractory elevated intracranial pressure:
  - Metabolic suppression (e.g., burst suppression targeted EEG-guided barbiturate coma). Lowers oxygen consumption of the brain by approximately 50% and thus decreases cerebral blood flow and cerebral blood volume, especially if autoregulation is preserved. No proof of improved outcome; risk of cardiovascular depression, risk of infections);
  - Hypothermia (e.g., 34 °C; lowers oxygen consumption of the brain and thus cerebral blood flow and cerebral blood volume). Evidence comes mainly from animal experiments; there is no solid beneficial evidence in humans, but an elevated risk for cardiovascular compromise and infections. In a recent multicenter study, it could be shown that in patients with an intracranial pressure of more than 20 mmHg, therapeutic hypothermia plus standard of care to reduce intracranial pressure did not result in better outcomes than standard care alone [61]. Therefore, therapeutic hypothermia is not recommended by the newest guidelines [2];
  - Decompressive craniectomy (DC) for selected cases only as DC is a last-resort treatment for severe refractory ICP, reducing ICP and mortality while increasing incidence of unfavorable outcome at six months [62];
  - Hypnocapnic ventilation (rescue therapy only; high risk of cerebral ischemia).

An interesting observation concerns the influence of increased intra-abdominal pressure (IAP) on ICP. It appears that intra-abdominal hypertension (IAH) transmits to the intracranial compartment, leading to an increase in ICP there. Conversely, it could be shown that decompressive laparotomy in patients with refractory ICP increases could lower ICP in patients with IAH [63]. 

#### 4.2.5. Lund Concept: Background and Clinical Application

The Lund concept, an ICP and volume targeted concept, is a therapeutic approach that focuses on the reduction of ICP by decreasing intracranial volumes [64]. This theory assumes that by reducing CPP, there is a reduced risk of promoting vasogenic edema and, therefore, less risk of elevating ICP. The concept was proposed by Asgeirsson et al. in 1994 [45]. In contrast to the previously described ’traditional’ therapeutic approaches, it emphasizes a reduction in microvascular pressures to minimize cerebral edema formation. Cerebral edema occurs due to vasogenic brain edema allowing for leakage of large molecules, such as albumin from blood vessels through the damaged blood–brain barrier. Water follows the large molecules (e.g., albumin) by osmosis into the extravascular compartment. This causes compression of and damage to brain tissue. The goals of the Lund concept are to preserve a normal colloid osmotic pressure by infusion of albumin and correction of anemia, to reduce capillary hydrostatic pressure by medical control of the blood pressure, and to reduce cerebral blood volume by vasoconstriction [65]. 

This is achieved by a flat head position, sedation, strict control of systemic hypertension, avoidance of neuromuscular blockade, hyperventilation, osmotherapy, and barbiturate coma [66]. 

The concept therefore includes:

1. Reduction of stress and brain metabolism by analgosedation with low-dose thiopental (0.5–3 mg/kg/h), use of beta-1-antagonist metoprolol, and use of alpha-2-agonist clonidine;
2. Reduction of hydrostatic capillary pressure with metoprolol and clonidine;
3. Maintenance of colloid-oncotic pressure, control of fluid balance with blood/albumin transfusions and use of furosemide.

Targets:

- ICP <20–22 mmHg;
- CPP 50–70 mmHg.

While the pathophysiological base of this concept is attractive, so far, no larger studies comparing the Lund concept versus other treatments exist. There are two small randomized trials (one with 68 and the other with 60 patients) comparing a CPP-targeted approach to a modified version of the Lund concept, which demonstrated a superior outcome with the Lund approach [67,68]. However, a systematic review published by the Cochrane Database of Systematic Reviews in 2013 did not establish evidence in favor to the Lund concept [69]. Since its introduction, the current concept does not differ much from the one originally presented, except that the venoconstrictor dihydroergotamine has been omitted. Dihydroergotamine was initially used in patients with a refractory, life-threateningly high ICP, but its use was discontinued because of potential adverse vasoconstrictor effects. Main criticisms arise from the fact that the Lund approach deemphasizes the effect of secondary cerebral ischemia and contradicts the common treatment goal of cerebral blood flow optimization by augmentation of cerebral perfusion pressure. Previous work demonstrated that low CPP causes reduction in cerebral blood flow and predisposes the injured brain to cerebral ischemia and infarction [49]. Within the range of autoregulation, a low CPP is associated with increased ICP through compensatory vasodilation in response to decreased perfusion pressure [49]. Studies associated with use of microdialysis, jugular venous oximetry, and brain tissue oxygen saturation (PbO_2_) have found that raising CPP above 60 mmHg may avoid cerebral oxygen desaturation in the injured brain that shows signs of ischemia [50,51,52,53]. Furthermore, use of albumin and steroids contradicts the findings in the SAFE and CRASH trial, respectively [59,70]. In conclusion, the Lund concept has not gained widespread acceptance outside of Sweden. This is due to very limited evidence of superiority compared to a CPP-targeted protocol and several controversial issues. Moreover, over the last decade, a certain convergence between the two concepts as a target CPP of 60–70 mmHg in the Lund concept and not above 70 mmHg in the CPP-targeted protocol has been observed [71].

### 4.3. Advanced Bedside Physiological Monitoring for Tailored Management of Cerebral Perfusion Pressure

As described above, the pathophysiology of TBI is highly heterogeneous, as it is influenced by the patient, the type of injury, and the treatment given. Thus, a one-size-fits-all management strategy is unlikely to be the optimum. More precise understanding of the influencing factors might allow for a better definition of patient-specific targets and better-tailored therapies.

Increased ICP, for example, is not a diagnosis by itself but rather a symptom resulting from different, usually coexisting processes. They include intracranial mass lesions, increased CBV either resulting from disordered autoregulation with vascular engorgement and/or excessive metabolic demand and/or hypercapnia, vasogenic or cytotoxic brain edema, or impaired CSF reabsorption.

Methods for more detailed identification of pathophysiological derangements were emerging in the past two decades using aggregated parameters derived from multiple monitoring techniques. CPP solely provides information on the driving pressure for blood flow through the cerebral circulation. Relevant downstream metabolic events can currently be detected only using probes for the measurement of jugular bulb oximetry (SjVO_2_), brain tissue partial tension of oxygen (PbtO_2_), and microdialysis, typically inserted through a common insertion bolt. Moreover, autoregulation assessment, registration of continuous electroencephalography (EEG) or its surrogate bispectral index (BIS), and different imaging modalities (e.g., transcranial doppler ultrasound) can be used to obtain additional information and potentially allow for individual targeted interventions. All these techniques generally provide indirect information about pathological processes with several limitations [72,73]. However, they have been used rarely, even in the most specialized neurological ICUs [74]. 

#### 4.3.1. Jugular Bulb Oximetry, Arteriovenous Difference in Lactate

Cerebral oxygen delivery (DO_2_) is the product of cerebral blood flow (CBF) and arterial oxygen content. In the healthy individual, the cerebral metabolic rate (CMRO_2_) is coupled to CBF such that when CMRO_2_ increases, CBF increases to match demand. The extraction ratio between arterial and venous blood remains constant. This is not the case in patients with head injury, in whom 50% or more exhibit evidence of defective cerebral autoregulation and subsequent uncoupling of CBF from CMRO_2_. Oxygen content is proportional to the saturation and therefore, the difference in arterial–venous (a–v) content of blood can be determined by the difference in SaO_2_ and SjVO_2_. SjVO_2_ is therefore a function of the arterial oxygen saturation, CBF, and CMRO_2_. In the situation where CMRO_2_ increases without a concomitant increase in CBF, the a–vO_2_ difference will rise in conjunction with cerebral oxygen extraction. 

Continuous monitoring of SjVO_2_ is performed though a fiberoptic catheter placed normally in the right internal jugular vein as cortical areas predominantly drain via the right jugular vein [75]. Another option would be to insert the catheter into the vein with the greatest flow. This could be accomplished using ultrasound to visualize the dominant vein. The tip of the catheter is advanced in the bulb in order to prevent extracranial blood contamination. Continuous monitoring of SjVO_2_ allows for estimation of the balance between global cerebral oxygen delivery and utilization [76,77] and reflects cerebral oxygen deficit. Moreover, cerebral ischemia by means of cerebral lactate production can be detected by an increase in arterovenous difference in lactate (AVDL) if systemic and jugular venous lactate is measured in parallel. Under conditions of stable cerebral metabolism, changes in SjVO_2_ reflect changes in CBF [78]. SjVO_2_ therefore has a prognostic value, and a decrease in SjVO_2_ below 55% is associated with a poor outcome [79]. Chieregato et al. demonstrated in 2002 that a CPP level below 60 mmHg was associated with abnormal arteriovenous lactate difference (AVDL) and SjO2 [80]. Thus, SjVO_2_ helps to determine the optimal CPP required for the maintenance of cerebral oxygenation. There are several limitations to being a global measure with limited sensitivity to regional changes [81]. SjVO_2_ does not decrease by <50% until more than 13% of the brain becomes ischemic [82]. 

Based on the Fick principle, cerebral oxygen consumption can be calculated by:

CMRO_2_ = CBF × (arterial O_2_ content − jugular venous O_2_ content), (1)

where:

CaO_2_(CvO_2_) = (Hb × % saturation × 1.34) + (arterial or venous O_2_ tension × 0.003). (2)

The difference between the venous and arterial oxygen saturation is used to interpret the cerebral oxygen requirements and to guide therapy. 

**Figure 2:**
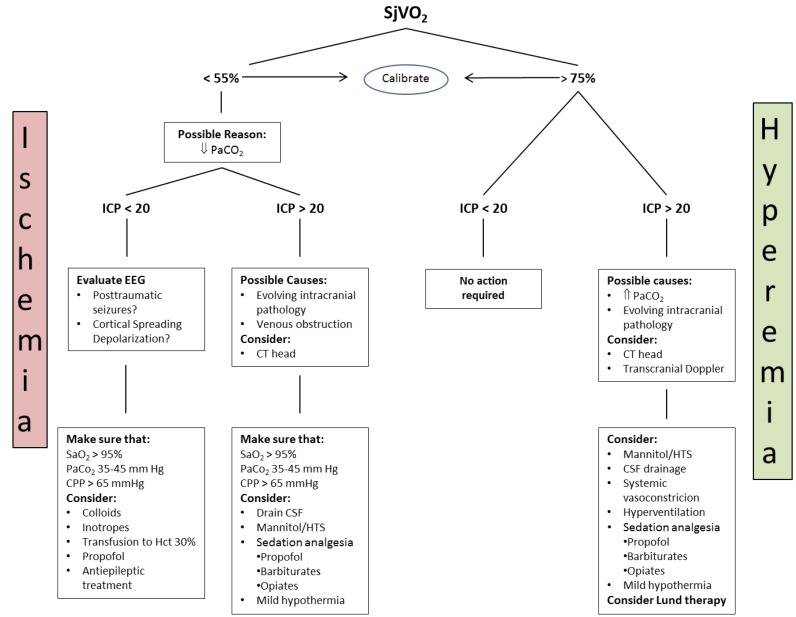
Interpretation and management of an abnormal SjVO_2_ (HTS = hypertonic saline). Adapted from White, H.; Baker, A. *Can. J. Anesth*. **2002**; *49*, 623–629 [83].

A proposed algorithm to manage abnormal SjVO_2_ was published by White and Baker in 2002 [83] (Figure 2).

As SjVO_2_ measurement is cumbersome due to technical reasons, it is not used very frequently in clinical routine. 

#### 4.3.2. Brain Tissue Partial Tension of Oxygen (PbtO_2_) 

Brain oxygen tension (PbtO_2_) can continuously be measured by an invasive probe with sensor using polarographic Clarke type electrode or fiberoptic technology [84]. PbtO_2_ provides a continuous (however very localized) measurement of extracellular oxygen tension as an indicator of the adequacy of oxygen delivery. It results from the balance between oxygen delivery and consumption, and the cerebral metabolic rate of oxygen (CMRO_2_). Normal PbrO_2_ is in the range of 35–50 mmHg [75]. Ischemic thresholds between 5 and 20 mmHg have been suggested [85,86,87]. Reduced PbtO_2_ has been associated with a poor outcome after neurotrauma [88]. However, PbtO_2_ is further modulated by oxygen diffusion [89]. Therefore, e.g., in pericontusional tissue, diffusion of oxygen is affected not only by tissue and endothelial edema but also by microvascular collapse, which increases the mean intercapillary distance for diffusion, reducing average oxygen tension [90]. Defining adequate target values for PbtO_2_ is therefore difficult. Values below 20 mmHg are typically accepted thresholds for inadequate oxygen supply and are associated with worse outcome after TBI [91]. Therapeutic approaches that aim to return PbtO_2_ to normal levels include increase of arterial pressure and, thus, CPP, increase of arterial oxygen tension (normobaric hyperoxia induced by increasing inspired oxygen fraction, FIO_2_) and/or increase of carbon dioxide partial pressure leading to an enhancement in CBF [92,93]. Use of PbtO_2_ may help with individualization of CPP. A number of studies have examined the relationship between CPP and cerebral oxygenation. After TBI, PbtO_2_ increases with CPP, and the ceiling of this effect is higher in the areas of focal ischemia. The increase in PbtO_2_ relative to an increase in arterial PO_2_ is termed brain tissue oxygen reactivity. It is believed that this reactivity is controlled by an oxygen regulatory mechanism (CBF autoregulation), and that this mechanism may be disturbed after brain injury. Soehle and colleagues introduced the concept of ‘autoregulation’, defined as the ability of the brain to maintain PbtO_2_ despite changes in CPP [94], thereby identifying appropriate individual CPP targets. Following these findings, manipulation of PbtO_2_ by increase of PO_2_ or altering the CPP (by increase of MAP, decrease of ICP, or both) have been investigated with the view to therapy optimization and potential prognostication [95]. While some studies showed a relationship between CPP and cerebral metabolic rate of oxygen (CMRO_2_), Sahuquillo et al. could not demonstrate a direct relationship between CPP and PbrO_2_ [96]. The PbtO_2_ readings varied with both normal and supranormal CPP. Stiefel et al. demonstrated that although PbtO_2_-directed therapy led to improved outcomes, CPP was similar between the groups [97]. CPP and ICP are not surrogates for PbtO_2_. Evidence that guided therapy using brain tissue oxygenation in addition to ICP and CPP monitoring leads to better outcomes after TBI is also increasing [98,99,100,101,102]. 

#### 4.3.3. Cerebral Microdialysis

The technique of microdialysis enables sampling and collecting of small molecular weight substances via a thin (0.6 mm), fenestrated, double-lumen dialysis catheter that is inserted into the interstitium of the brain. Placement in the vulnerable area after TBI provides online analysis of extracellular/interstitial biochemical changes in, e.g., glucose (energy substrates), lactate, pyruvate (brain glucose metabolism), glycerol (cell membrane degradation), and glutamate (excitatory neurotransmitter). A high lactate:pyruvate ratio (LPR) is a marker of ischemia and/or diffusion hypoxia, leading to anaerobic glycolysis or—under normoxic conditions—mitochondrial failure (e.g., resulting from intracellular calcium overload). This indicates an energy metabolic crisis and is an independent predictor of mortality [103]. A reduction in LPR might be a sign of beneficial treatment effects, e.g., from hyperoxia and/or enhanced MAP as imaging studies demonstrated improvements in CMRO_2_ and reversal of pericontusional cytotoxic edema with normobaric hyperoxia [104]. Moreover, microdialysis demonstrates that low brain glucose (<3 mmol/L), influenced by arterial blood glucose (<6 mmol/L) leads to elevated LP and, lactate:glutamate ratio, and brain lactate:glucose ratio is lowest at brain glucose levels above 5 mmol/L, demonstrating that not only hyperglycemia but also hypoglycemia is detrimental to brain energy metabolism [53]. Conversely, infusions with hypertonic lactate show a clear cerebral glucose sparing effect but only in patients with a pathological LPR [105]. Low glucose resulting from decreased delivery by decreased CBF or increased consumption (e.g., epileptic discharge) as well as an increase in the LPR resulting from anaerobic glucose metabolism due to ischemia above established thresholds is associated with poor outcome in neurotrauma [106,107]. Glutamate is the major excitatory amino acid in the brain. Derived from glucose, glutamate is a surrogate marker for neuronal oxidative metabolism under normal conditions. Astrocytic uptake keeps brain interstitial glutamate levels low by converting released glutamate into glutamine, which is reconverted to glutamate by the neurons. Because this cycle requires energy, an impaired energy metabolism may lead to low interstitial glutamine/glutamate ratios [108]. In the acute post-TBI phase, neuronal depolarization induced by an intracellular Ca_2_+ influx is followed by an increase in interstitial glutamate, higher anaerobic glycolytic activity, and increased lactate production. Uncontrolled release as observed in TBI, on the other hand, leads to excessive calcium influx into brain cells via glutamate-mediated ion channels [109]. Glutamate has been considered an early marker of cerebral ischemia [110]. Glycerol is the final product of the enzymatic degradation of cell membrane triglycerides, and its presence indicates a loss of cellular structural integrity [111]. Glycerol is also considered a reliable indicator of tissue ischemia that has progressed to a stage that involves further cell damage [112]. Cerebral microdialysis glycerol increases relatively slowly during energy metabolism failure and remains elevated for some time upon normalization [112]. Biochemical changes of microdialysis occur before low CPP is detectable [113]. 

#### 4.3.4. Clinical Use of Cerebral Microdialysis

Cerebral microdialysis can be used to determine the optimal level of CPP in patients with TBI. Cerebral microdialysis analyses can reveal alarming levels of LPR and glucose. Ischemia is defined by the combined CMD criteria of LPR > 40 and glucose < 0.2 mmol/L. In persistent ischemia, LPR may exceed 40 and often plateaus at a value between 80 and 120. Lactate:pyruvate ratios as high as 500–1000 have been documented in cases of impending brain death [114].

Studies using CMD in normal and pericontusional brain tissue have shown significantly higher LPR values in patients with ICPs >20 mmHg, with most pronounced values when ICP exceeds 30 mmHg. These studies have also demonstrated a significant increase in LPR in pericontusional brain tissue with decreasing CPP due to the loss of CBF autoregulation [115]. Changes in LPR may precede the onset of intracranial hypertension, and increased pericontusional LPR is significantly associated with CPP [116]. Moreover, low PbtO_2_ and a high LPR are more frequently observed with high ICP and low CPP [116]. Nordstrom demonstrated that lactate in the injured brain increased both when CPP was less than 50 mmHg as well as more than 70 mmHg [112]. Stahl et al. showed that microdialysis constituents could be kept in a normal range only if CPP was more than 50 mmHg, and significant ischemia developed below this threshold [117]. Two studies showed that although increased CPP with vasopressors improved CBF and oxygenation, it failed to demonstrate any significant change in microdialysis indices [93,118]. Increased LPR values have also been linked to different causes of alterations in oxygen use, such as oxygen diffusion barriers, mitochondrial dysfunction, and increased metabolism of glucose [119]. The lactate/glucose ratio may also be an indicator of increased glycolysis [109]. Intracerebral CMD glucose concentration may also decrease after insulin therapy, even if blood glucose levels remain unaffected. This observation is supported by data from studies of TBI and patients after subarachnoidal hemorrhage, which have shown that intensive insulin therapy results in a net reduction in CMD glucose, an increase in cellular distress markers, and an oxygen extraction fraction near ischemic levels, even in the absence of profound hypoglycemia [120,121]. In patients with severe TBI, tight systemic glucose control is associated with an increased prevalence of brain energy crises and low cerebral extracellular glucose, which in turn correlates with increased mortality [122]. Administration of insulin in these patients should therefore be carried out with caution. 

As described above, CMD has shown an increase in excitatory amino acids, particularly glutamate after TBI. Transient critical reductions in CPP may be associated with increases in glutamate, indicating that neuronal populations are as vulnerable to alterations in CPP as they are to ischemia. In a few cases, glutamate will increase before CPP drops. It is possible that glutamate increases at a higher CPP threshold and that its release leads to cellular swelling and increased ICP [123]. 

#### 4.3.5. Cerebral Microdialysis and Seizures, Cortical Spreading Depolarization 

Posttraumatic seizures have been found to affect 22% of patients suffering from TBI and are associated with greater and prolonged intracranial hypertension and an increased mortality rate. Prompt recognition and treatment is therefore essential. However, electrographic seizures without any clinical signs account for over 50% of seizures in the intensive care unit. Cerebral microdialysis allows for detection of LPR, glutamate, and glycerol increase when electrographic seizures occur [109]. High LPR persists longer and occurs more frequently in patients with electrographic seizures [108,124]. Prolonged increases in CMD-measured glutamate levels are associated with repetitive seizures that occur over many hours. Seizures are accompanied by increases in mean arterial blood pressure and CPP. This may be a physiological response attempting to meet the brain’s greater metabolic demands. However, increased CPP may in turn contribute to further cellular injury in the condition of decreased intracranial compliance and alterations in the blood–brain barrier. It is therefore possible that an increase in CPP in the context of impaired pressure autoregulation may lead to hyperemia, and cellular injury may occur despite adequate CPP [116]. 

Cortical spreading depolarization (CSD) was described for the first time in 1944. Cortical depolarization waves spread across the cortical surface at 2–3 mm/min. Marked glucose depletion occurs during CSD in proportion to the number of depolarizations [125]. This electrical disturbance occurs in 50% of patients with severe TBI and is an important cause of early ischemia in TBI and SAH patients [108,126]. Some authors have reported that CSD coincides with a 200% increase in glucose use in the injured cortex and the ipsilateral hippocampus [127,128]. Another study showed a progressive decline in CMD-measured glucose associated with recurrent depolarizations in patients with head injuries. Under these particular conditions, a vicious cycle is established; pre-ictal discharges (PID) deplete the residual glucose pool and thus increase the probability of additional PIDs. Transient glucose depletion appears to be caused by a combination of increased use and reduced vascular delivery, as in the case of strict serum glucose control that was previously described in this article.

There is a transient increase in lactate that can be attributed to a temporary elevation in the rate of glycolysis [129]. In most animal models, CSD is associated with the transient dilation of superficial arterioles followed by sustained constriction. Microvascular vasoconstriction leads to parenchymal hypoperfusion during the period of maximum metabolic demand. It is more likely that neurovascular coupling is disrupted and that profound neuroglial depolarization causes swelling that constricts the capillary bed [130]. In some patients with few initial CSDs followed by repeated episodes of periinfarct depolarization, depolarizing episodes may be related to brain tissue hypoperfusion and expansion of the ischemic penumbra, which potentially expands the core infarct zone and increases the final infarct volume [126,129,131]. 

### 4.4. Assessment of Autoregulation

Autoregulation is a physiological mechanism that maintains adequate cerebral perfusion in the presence of blood pressure changes. With normal autoregulation, the diameter of cerebral vessels adjusts for alterations in arterial pressure (e.g., vasoconstriction as response to an increase in arterial pressure). As vasoconstriction reduces cerebral blood volume, it might decrease an elevated ICP.

Influence of MAP variations on ICP can therefore be used for online real-time assessment of cerebrovascular autoregulation, e.g., by calculating the pressure–reactivity index (PRx), a correlation coefficient between ICP and arterial pressure. The CPP for which the PRx is a minimum represents a state of optimum autoregulation. CPP-based managements that target this level have been shown to be associated with better outcomes and might prevent cerebral hypoperfusion as well as excessive cerebral blood flow [132,133].

In severe TBI, autoregulation can be impaired or totally lost. Moreover, autoregulation can be impaired regionally and not be detected by the PRx, which is a global parameter. Alternative measures, e.g., based on assessment of blood flow or brain tissue oxygen reactivity, have the opposite limitation of restricted global spatial coverage. Prospective evidence from clinical studies is urgently needed before definitive conclusions can be drawn.

### 4.5. Simultaneous Multimodal Monitoring for Individualised Management

Simultaneous use of several monitoring modalities allows for provision of information to define individual patient-specific treatment targets and thresholds and to perform cross-validation of the physiological state of the injured brain [72]. Discordant downstream markers of compromised cerebral metabolism, although potentially imposing a dilemma in terms of treatment compromise, might sometimes stimulate the search for and approach to less well-recognized reasons for energy failure, such as diffusion hypoxia mitochondrial dysfunction and low cerebral glucose levels [134,135,136,137]. 

Current multimodal monitoring generates, however, giant amounts of data, which have to be sorted and summarized for daily use in order to enable clinicians to extract information needed to optimize treatment. Advances in monitoring will probably also depend on advances in neuroinformatics and data analysis. Computer visualization techniques offer a promising way to reduce complex datasets to a form that can be interpreted by clinicians and have been applied in various areas, including investigation of the cumulative burden of intracranial hypertension and assessment of autoregulation [138,139]. Such complex multidimensional problems are not new outside medicine, and other so-called big data techniques will very likely find increasing application in the intensive care of patients with TBI.

## 5. Specific Considerations in Traumatic Brain Injury in the Elderly

There is a clear association between older age and worse outcomes, at least in part, attributed to age-related comorbidities, and use of medications for its treatment [140,141,142,143]. 

Frequently used anticoagulant and antiplatelet drugs increase the risk for hemorrhage or can worsen the evolution of intracerebral traumatic lesions [144]. The fact that GCS might underestimate the severity of TBI potentially increases the threshold to refer patients to specialist centers [145,146].

Moreover, as mentioned above, age-related comorbidities promote secondary brain damage and reduce (but not abolish) plasticity and neural repair in the elderly, compromising the success of rehabilitation [147]. The monitoring and treatment of comorbidities is therefore as important as management of TBI itself in determining outcome. That is, treatment of drug-induced coagulopathy with reversal of anticoagulant or antiplatelet therapy is crucial in impending or present intracranial hemorrhage. Epileptic seizures have to be detected early and met with prompt treatment. However, the optimum therapy and length of seizure prophylaxis in this population is still not clear. As mentioned above, in older patients, a lower ICP threshold is already associated with poor outcome. This might reflect a greater vulnerability of the aged brain and/or may indicate a worse brain injury as atrophy and increased CSF space allows for a more pronounced lesion expansion and brain edema before ICP increases. This would provide a rationale to investigate whether a reduced threshold for ICP control could be beneficial in this population. However, as lower frequencies of ICP increases in older people can be observed and insertion of intracranial probes might be riskier, especially in patients under anticoagulant and antiplatelet drugs, a conservative approach towards ICP monitoring in these patients has to be considered. 

Elderly patients might also have compromised autoregulation because of chronic arterial hypertension. This shifts the autoregulatory curve to the right towards higher arterial pressure. Available data demonstrate better survival in patients older than 55 years, particularly with chronic arterial hypertension, if CPP thresholds are higher compared to those of younger patients [32].

It has to be stressed that an unfavorable outcome in older patients could also be a self-fulfilling prophecy. First of all, current concepts of TBI management are derived from younger patients with high-velocity injuries. Therefore, translation of these findings directly to the treatment of older patients with different injury mechanisms might not be justified or might even be wrong. Facing an increasing proportion of older patients with TBI, there is an urgent need to find and establish tailored management strategies for these patients. Different literature demonstrates that older patients (not only with head injuries) are prone to suboptimal care, including delayed imaging, are assessed/treated more commonly by junior medical staff, and have a reduced likelihood to be transferred to a neurotrauma center [74,148]. Nevertheless, when older patients are treated aggressively and promptly after admission to the ICU, favorable outcomes are seen in 39% of patients aged 60–69 years, suggesting that for carefully selected patients, this approach can also be justified [2,24].

## 6. Conclusions

Acute traumatic brain injury is a very complex condition that can result in physical, cognitive, social, emotional, and behavioral symptoms, and outcome can range from almost complete recovery to permanent disability or death. Heterogeneity of TBI as well as concomitant injuries and co-morbidities preclude a one-size-does-fit-all treatment approach. Moreover, bringing patients with brain injuries back to an enjoyable life should be the ultimate goal of any treatment. It is important to realize that in the treatment of TBI, a not exceptional “medical success” may result in a social catastrophe. Therefore, it is crucial to select the right patient for the right and most promising treatment, and refrain from interventions aiming at survival only. Unfortunately, there is still a lack of understanding and specifically targeting all the adverse pathways induced by the brain damage. Better understanding of the damage mechanisms and new approaches to neuroprotection restoration may offer better outcomes for millions of survivors of TBI.

## Figures and Tables

**Table 1:** Escalating options to treat elevated intracranial pressure.

Intervention	Effect	Pot. Benefit	Pot. Risk
(Deepening of) Analgosedation, Barbiturates	Decrease of brain metabolism and therefore oxygen consumption	Decrease in CBF, CBV and ICP	Adverse effects of long-term sedation, impaired neurological assessment
Osmotherapy with Mannitol	Reduction in brain tissue volume, increase in CBF	Decreases ICP, no improvement of tissue oxygenation	Fluid overload, osmotic diuresis leading to hypovolemia, hyperosmolarity, rebound brain edema
Osmotherapy with hypertonic saline	Reduction in brain tissue volume, increase in CBF	Decreases ICP, improvement of tissue oxygenation	Fluid overload, osmotic diuresis leading to hypovolemia, hyperosmolarity
CSF drainage	Allows for expansion of brain tissue by reducing CSF volume	Lowers ICP	Transcerebral ventricular drainage required, risk of ventriculitis, meningitis
Hyperventilation	Cerebral vasoconstriction leading to reduced CBV	Lowers ICP if autoregulation is intact	Reduction in CBF leading to brain tissue ischemia
Decompressive craniectomy	Allows for expansion of the edematous brain	Decreases ICP, may improve tissue perfusion	Aggravation of brain edema, unfavorable outcome after 6 months

ICP: Intracranial pressure, CBV: Cerebral blood volume, CBF: Cerebral blood flow, CSF: Cerebrospinal fluid.
